# DNA Methyltransferases: From Evolution to Clinical Applications

**DOI:** 10.3390/ijms23168994

**Published:** 2022-08-12

**Authors:** Victor M. Del Castillo Falconi, Karla Torres-Arciga, Genaro Matus-Ortega, José Díaz-Chávez, Luis A. Herrera

**Affiliations:** 1 Instituto Nacional de Cancerología (INCAN), Avenida San Fernando No. 22, Sección XVI Tlalpan, Ciudad de México 14080, Mexico; 2 Unidad de Investigación Biomédica en Cáncer, Instituto de Investigaciones Biomédicas, Universidad Nacional Autónoma de México (UNAM), Coyoacán, Ciudad de México 04510, Mexico; 3 Departamento de Bioquímica, Facultad de Medicina, Universidad Nacional Autónoma de México (UNAM), Coyoacán, Ciudad de México 04510, Mexico; 4 Instituto Nacional de Medicina Genómica, Periferico Sur 4809, Arenal Tepepan, Tlalpan, Ciudad de México 14610, Mexico

**Keywords:** 5-methylcytosine, cancer biology, DNA methylation, DNA methyltransferases, DNA methylation, DNMTs isoforms, epigenetics

## Abstract

DNA methylation is an epigenetic mark that living beings have used in different environments. The MTases family catalyzes DNA methylation. This process is conserved from archaea to eukaryotes, from fertilization to every stage of development, and from the early stages of cancer to metastasis. The family of DNMTs has been classified into DNMT1, DNMT2, and DNMT3. Each DNMT has been duplicated or deleted, having consequences on DNMT structure and cellular function, resulting in a conserved evolutionary reaction of DNA methylation. DNMTs are conserved in the five kingdoms of life: bacteria, protists, fungi, plants, and animals. The importance of DNMTs in whether methylate or not has a historical adaptation that in mammals has been discovered in complex regulatory mechanisms to develop another padlock to genomic insurance stability. The regulatory mechanisms that control DNMTs expression are involved in a diversity of cell phenotypes and are associated with pathologies transcription deregulation. This work focused on DNA methyltransferases, their biology, functions, and new inhibitory mechanisms reported. We also discuss different approaches to inhibit DNMTs, the use of non-coding RNAs and nucleoside chemical compounds in recent studies, and their importance in biological, clinical, and industry research.

## 1. Introduction

DNA methylation is an essential epigenetic mark that living beings have used to survive in different ambient conditions. For example, in prokaryotes, it is used to differentiate their own DNA from foreign DNA and avoid endoreduplication [1]. Moreover, in eukaryotes, DNA methylation is used to silence DNA fragments and whole chromosomes, program cell differentiation, and avoid errors in DNA cell segregation [2,3,4]. In addition, DNA methylation is a promising molecular tool to cause changes in living beings’ phenotypes in an epigenetic way, for example, in plants or mushrooms metabolites production, reproduction, differentiation, or clinical use to treat diseases [2,3,4,5]. In mammalians, there are several mechanisms to regulate the expression or the gene dose of DNMTs. In cancer, these mechanisms have been shown to be the product of alternative splicing isoforms and non-coding RNAs that regulate DNMTs mRNA expression [6,7]. Interestingly, these regulatory mechanisms are affected in cancer, changing dose in initiation, progression, and metastasis. In this sense, several compounds inhibit DNA methylation and are probed to combine chemotherapies in cancer treatments [8,9]. Therefore, DNA-methyltransferases (DNMTs) are an exciting subject for researching the capacity to manipulate the living cells’ phenotypes.

The MTases family catalyzes DNA methylation, and it is a conserved mechanism from archaea to eukaryotes, from fertilization to every stage of development and from early stages of cancer to metastasis [6]. However, to do or not to do, “methylate or not methylate DNA” is an important question that MTases need to answer. Many examples and many inhibitory mechanisms throughout evolution have been reported. For instance, in prokaryotes, MTases methylate host DNA, and endonucleases digest foreign unmethylated DNA [10]. MTases methylate the X chromosome in eukaryotes and repress gene expression [11]. During development, MTases need to be silenced, and as a result, DNA methylation is absent [12]. Many different inhibitory mechanisms have been reported in cancer, such as non-coding RNAs that target MTases and overexpression of inactive MTases isoforms product of alternative splicing [13]. These studies suggest that methylate or not methylate DNA is a precise decision to survive in different ambients and developmental states.

This work focused on DNA Methyltransferases, their biology, their functions, and their new inhibitory mechanisms reported. Many studies have focused on DNMTs because they have an important role in the epigenetic regulation of living beings [14]. Other interesting opportunity areas of research in MTases could be in treatments of human and plant parasites, the reproductive interest and plants, fungi metabolites, and other cancer or psychiatric disorders treatments [15,16]. Interestingly, studies in DNMTs expression have focused on social context. Arthropod studies have shown that different population statuses have different quantities of DNMTs, suggesting a role in DNA methylation and the hierarchical social order in the species [17]. These are several examples of DNMTs researchers’ interest and findings at this time. Some regulatory mechanisms in cancer have been reported as the competence of isoforms without catalytic domain or non-coding RNAs expression to target DNMTs. And also, the impact of expression of DNMTs is a good topic as a biomarker and target in cancer [18]. DNMTs are essential in living beings’ evolution. Understanding them could significantly impact several areas to develop new molecular tools; however, more research is necessary.

## 2. DNA Methyltransferases

### 2.1. The Structure of MTAses

DNA methyltransferases (MTases) are conserved in living beings acting in orchestra with other epigenetic players. They have positioned themselves with a few exceptions as the main transcription regulators. MTases are a group with methyltransferase activity; they have evolved in different orthologs, but all have the methyltransferase domain and a DNA target recognition domain. Living beings have conserved MTases to survive the different and dynamic ambient conditions [2,3,4,5,19].

**Figure 1:**
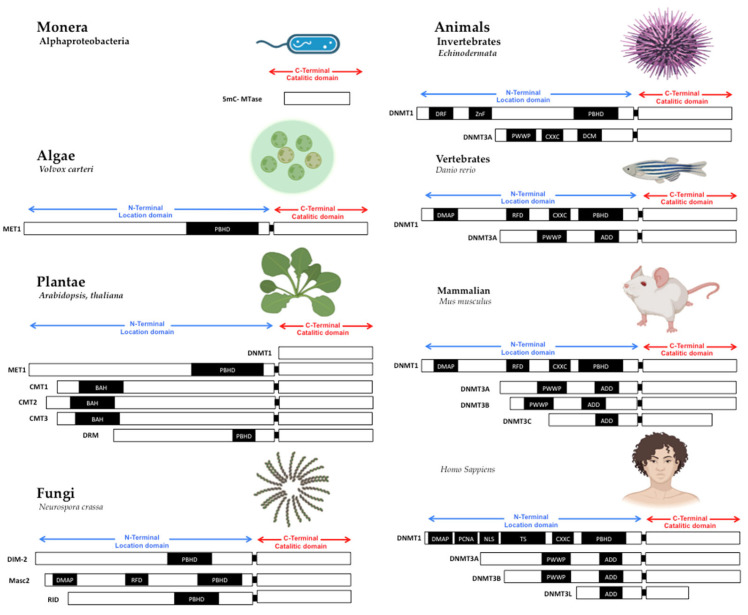
Structure of DNA methyltransferases. The DNMTs family is the DNA methylator in living beings. DNMTs have two domains: catalytic domain in the carboxy-terminal extreme, with the conserved catalytic motives, and location domain in the amino-terminal extreme, with the location and interaction chromatin motives. Monera has the catalytic domain mtase, Alphaproteobacteria. Protists and Algae are composed of MET1, CMT3, and DRM2 protein paralogs; MET1 is an example present in Volvox carteri. Fungi are composed of the protein paralogs: DIM-2, Masc1 and 2, and RID; the example is Neurospora crassa. In animals, Invertebrates, the protein paralogs are DNMT1 and DNMT3, in Echinoderma; Vertebrates, Fishes, DNMT1, and 3, zebrafish has 8 DNMT3 mammalians, the protein paralogs are: DNMT1 and DNMT3A/B/C in Mus musculus, and DNMT1, 3A/B/L in Homo sapiens. Abbreviatures: CD (chromo dominio), DMAP-1, binding domain (DMAP); motif to interact with PCNA (PCDNA); Nuclear localization Signal (NLS); Targeted Site (TS); Motif to Cys-X-X-Cys amino-acids, with zinc fingers (CXXC), Protein Binding Homeo Domain (PBHD); the motif of interaction with pro-trp-trp-pro (PWWP), and ATRX, DNMT3, DNMT3L domain (ADD). Note: Created with Biorender.com, accessed on 21 July 2022.

The reaction mechanism of MTases catalyzes DNA methylation in adenine or cytosine bases is known. All MTases interact with the cofactor S-adenosyl methionine (AdoMet) to transfer a methyl group and produce S-adenosyl-l-homocysteine (AdoHcy) and methylated DNA [20]. Moreover, another characteristic of all MTases is that they have three protein domains (in the carboxy-terminal domain for DNMTs): The adoMet binding domain, which interacts with AdoMet to obtain the methyl group; a target recognition domain (TRD), which recognize a short sequence of DNA to be targeted for methylation, and the catalytic domain, which transfers a methyl group to AdoMet to the targeted nucleotide [20] (Figure 1). These domains are the set of MTases, which beings have conserved in almost all species of living beings.

The primary sequence of MTases is essential to methyltransferase activity, but the shape is also crucial. The structural conformation domains and different motifs of MTases have been reviewed in other works [6,7], where domain or domains conformed MTases in all cases. First, in the amino-terminal domain, some motifs interact with CpG sequences as the CXXC domain in DNMT1 [21,22,23]. Still, without the CXXC motif, DNMT1 can’t actively interact with the PCNA motive to serve in DNA replication [24,25]; the PWWP motif in DNMT3A and DNMT3B, which interacts with chromatin proteins and localizes them in centromeric and pericentromeric chromatin [11,26]. The shape and the amino acid primary sequences of MTases are essential to be conserved in the catalytic domain in all living beings. The variable part on TRD is also evolving to give specificity to DNMTs duplications [25]. 

### 2.2. DNA Cytosine 5′MTases Biology

MTases had been shown duplicated in different taxa to give place to different paralogs in any species. MTases have redundancy functions, but MTases could influence the gene transcription specificity in the variation of living beings to their own ambient conditions. For example, in mammals, DNA methyltransferase 1 (DNMT1) was the first DNMT identified with DNA methyltransferase activity. DNMT1 is called maintenance DNMT because it methylates hemimethylated DNA through the cell cycle to maintain the epigenetic memory in differentiated cells [11,26,27]. DNMT2 is the most widely distributed DNMT in living beings. DNMT2 has RNA methylation activity, and it is believed that DNMT2 was the first DNMT in life [2]. DNMT3 is a paralog gene of DNMT that has been duplicated many times in different organisms: DNMT3A, DNMT3B, and DNMT3L are the most frequent duplication events; however, it has been found more than one duplication event in zebrafish. DNMT3 isoforms can also interact between them as accessory proteins putting together a DNMT3 complex. In fact, DNMT3L binds to DNMT3A and DNMT3B and enhances their catalytical activity [28]. Recently, DNMT3C was described as a novel paralog of DNMT protein reported that originated from DNMT3B duplication. DNMT3C has been studied in mice, and it has been shown that it functions in retrotransposon methylation and other repetitive sequences. Interestingly, DNMT3C has a vital role in male fertility [29,30]. In summary, the MTases paralogs have evolved to have a specific role in epigenetic gene regulation in most of the species, and it could have consequences on adaptation.

DNA 5`cytosine methyltransferases have been reported in several species of the five kingdoms of life. The names used in every work depend on the life model studied; for example, in prokaryotes, it has been called MTases, whereas, in eukaryotes, they are called DNMTs. In algae and plants, DNMTs and chromomethylases are the names used, respectively (Table 1). DNA methylation is an evolutionarily conserved reaction, and it is conserved in the five kingdoms of life: bacterias, protists, fungi, plants, and animals; this is true with a few exceptions of species that have not detectable DNA methylation, such as *Drosophila melanogaster* [31,32], that has DNMT2 [33]. *Saccharomyces cerevisiae, Saccharomyces boulardii*, *Schizosaccharomyces pombe* [34], and *Caenorhabditis elegans* (Table 2) [35]. MTases have been positioned as an essential enzyme that regulates gene transcription; however, we can know that these examples are important and not the only factor involved in epigenetic control. Next, we described the MTases history in life kingdoms and the regulatory transcription mechanisms developed to control MTases.

First, the MTases sequences and functions have been found in viruses. Viruses have ribose [48] and deoxyribose methyltransferases [49,50]. MTases have been found in viruses, for example, LM21 prophage in *Sinorizhobium* [51] or the methyltransferase domain in rhinovirus and coronavirus that targets RNA cap [52,53], and ebolavirus that targets adenosine in RNA [54]. One mechanism exploited by some viruses is to induce the upregulation of mRNA of DNMTs in hepatitis, cirrhosis, and hepatocellular carcinoma, observed in the presence of hepatitis B virus [55,56]; Epstein Barr virus in gastric cancer [57,58,59]; herpesvirus LANA, in Kaposi’s sarcoma [60]; and interestingly in lymphoid cells, infected by human immunodeficiency virus 1 [61].

Prokaryotic MTases protect DNA from several ambiental conditions: against the virus, prokaryotic MTases have a role in the restriction-modification (RM) system, which consists in that MTases methylate host DNA and do not methylate foreign DNA [62]. It is to recognize their own DNA and to avoid DNA that could be lethal for the cell. It is, in fact, one of the first functions of DNA methylation. To differentiate internal DNA from external DNA. Therefore, MTases have an essential role in life evolution [1], especially in the cell cycle and regulation of gene transcription [63]. Several examples of archaea MTases have been studied; there are *Haloferax volcanii* [64], *Pyrocuccus abyssi* [65], *Pyrococcus horikoshii* [66,67], *Sulfolobus solfactaricus* [68], and *Thermococcus gammatolerans* [69]. In bacteria, examples include *Alphaproteobacteria* [36], *Borrelia burgdorferi* [70], *Escherichia coli* [71], *Helicobacter pylori* [72], and also *Mycobacterium* species [73].

In protists, we know that there are 5′ cytosine methyltransferases homologs because they have been reported by analysis in silico [37]; however, this has not been probed experimentally or either their function [37]. Probably, DNA methylation has an essential role in parasite infection, specifically in the adaptation to the host. Furthermore, it could have clinical importance to infection treatments because MTases expression could have changed during the different stages of life in protists parasites.

Algae and plants have DNA methylation and the same MTases homologs. In algae, MTases have been reported in the multicellular green algae. The MTase gene in *Volvox carteri*, Methyltransferase1 (*met1),* is a homolog of DNMT1 in mammals and is detected during the DNA replication fork. It has been localized in transposon CpG sequence methylation [38]. Nowadays, there are no reports of chloroplast having DNA methylation. There are several types of conserved MTases in algae and plants. They are called distinctive names: Methyltransferase 1 (*met1*) Chromomethylases (CMT), Domains Rearranged Methyltransferase (DRM). In algae, MTases are overexpressed during the sexual and asexual phases of the green algae *Boechera* genus [39]. These facts could suggest that Mtases probably have a role in different types of reproduction, from algae to plants. In this sense, species with more than one reproduction type could have differences in their own DNA methylation. And it could be involved in the formation of the sexual types of diversity evolution. However, studies are lacking on these issues.

MTases in plants such as *Arabidopsis thaliana* and *Salvia milthiorrhiza* act in gene methylation of non-coding RNAs and coding RNAs; also, in plants, MTases act in the phenomena of genomic imprinting, calling of epi-alleles [16,43,74]. Recently, several studies have been focused on the epigenetic control of commercial plants or products where DNMTs involve in cotton fruiting branch development [75]; they are also present in the globe artichoke, *Cynara cardunculus* var. *escolymus* development [76]; in the tomato *Solanum pennellii* [77], stress response and development; in legumes regulation of development and life cycle [78]; in production of oil palm by *Elaeis guineensis,* and plants detoxification of pesticides [79]. These studies have shown that DNMTs have an important role in industry; as markers of stress, or toxic conditions, in specific taxa of plants, they could be a valuable tool for the environment.

Fungi is a biological kingdom that has let us see the importance of metabolite production and the diversity of various types of metabolisms that have evolved to survive on every external condition. Although in fungi [80], homolog DNMTs have been poorly experimentally studied, DIM-2, DNMT1, DNMT5, and RID are present [43,80] and have a function in metabolism and secondary metabolite production: of xylanase, a plant enzyme function in cell wall degradation of *Humicola grisea* [41]; the production of cytochalasin E, an antibacterial and anti-angiogenic compound in *Aspergillus clavatus* [42], and the production of aflatoxin in *Aspergillus flavus* [81]. Fungi are a big group of living beings. They have several types of reproduction, metabolites, forms, growing, pathogenicity, and different clinical interest in life interactions. Mushrooms could be an exciting model to research the role of DNA methylation in infections or how they compete with other groups of mushrooms or plant colonization.

In animals, MTases are DNA methyltransferases (DNMTs), which have been reported in several functions on invertebrates and vertebrates. DNMTs are present in all animal phyla. DNMTs are functional in radial animals, echinoderms: starfish and sea urchins. Interestingly, not all DNMTs are present in echinoderms; they have only DNMT1 and DNMT3. Another intriguing enigma to research is why DNMT1 does not have a PCNA binding domain in starfish [82]. Another interesting example is the absence of DNA methyltransferases in Nematodes [83]; this is evidence that living beings can have optimal development and infectious independent of DNA methylation. However, other epigenetic changes such as histone modifications and non-codificant RNAs could be involved [45].

DNMTs are essential in the epigenetic memory of animals. Different types of memory in cells have been affected when DNMTs are inhibited. For example, early development is affected in the Oyster *Crassostrea gigas* treated with 5-azacitidine, an inhibitor of DNMTs catalytic activity [84]; on the other hand, memory consolidation of long-term memory neuronal system in mollusks is affected when *Aplysia* is treated with RG108, another compound that inhibits DNMTs. This treatment was applied for 24 h and resulted in amnesia until 48 h [85]. Moreover, interestingly, in arthropods, it has been proposed that DNMTs are involved in embryonic development [86], fecundity, and behavior because they have differences in DNA methylation and DNMTs expression patterns, for example, in the heads of the workers and gynes of the ant, *Solenopsis invicta*, DNA methylation and DNMTs expression of different embryos and adults changed eight-fold [44,87] and similar results were found in young workers compared with old workers and the queen from honey bees [17]; the excellent olfactory memory was interrupted by inhibiting DNMTs activity with the inhibitor of DNMTs, zebularine [88,89].

DNMTs are involved in vertebrate evolution and have been studied since embryonic development. In addition, it has been shown that DNMT3A leads the embryonic development [90]. For example, the role of DNMT3B in activating FOX2A, a transcription factor that participates in endoderm development [91]. Interestingly, several paralogs and isoforms products of alternative splicing that have a role in embryonic development and cancer have been recognized in vertebrates. Vertebrates have a diversity of DNMTs and internal transcription regulatory mechanisms that function in several conditions to express different phenotypes. 

In the zebrafish genome, 8 DNMTs have been recognized: one DNMT1, one DNMT2, and six DNMT3 [47], and they are regulated during embryonic development [92] and by temperature changes [3,93]. DNMT3A and 3B families are similarly regulated during embryonic development [94] and the sex of this animal, suggesting that these families have a similar transcription regulation control. It has been proposed that the diversification of DNMT3 occurred early during the evolution of vertebrates [92]. For example, 3AA is overexpressed and not 3AB for sex differentiation in *Oreochromis niloticus* [95]. Other examples in vertebrates are found in *Solea seleganensis,* which has five paralogs of DNMTs that are regulated differentially during development. In addition, it is a model where it has been demonstrated that DNMT expressions change with temperature and 5-aza-2′deoxycitidine treatment [46]. Moreover, in the Atlantic cod, the photoperiod has been associated with DNMTs expression [96].

In summary, several studies have shown the structural and functional role of DNMTs in vertebrates during development [97]. DNMTs in mammals, DNMTs have a role in embryonic development, but DNMTs produce alternative splicing [7,10]. It is a recent topic we will discuss in the next section because early embryonic development and cancer reprogramming cells have been associated. To this point, we have reviewed the DNMTs’ origin, function, and evolutionary history. In mammalians exists a lot of evidence about DNMTs as a product of alternative splicing that has an important role in cancer development and poor prognosis of the patients.

### 2.3. Regulating DNA Metiltransferases by Splicing Isoforms

To know the regulatory mechanisms, we have focused on splicing alternative isoforms. The alternative splicing process post-transcriptionally regulates DNMTs. DNMT1 and DNMT3A produce catalytically active isoforms, and DNMT3B produces catalytically active and inactive isoforms [98,99]. Ostler suggests that aberrant DNA methylation patterns in cancer result from overexpression of catalytically inactive isoforms of DNMT3B. Nowadays, fifteen DNMT3B isoforms have been reported. The main characteristics of DNMT3B isoforms are that they lack partially or the full catalytic domain and participate as negative regulators of DNA methylation. Therefore, they are overexpressed in several types of cancer.

**Figure 2:**
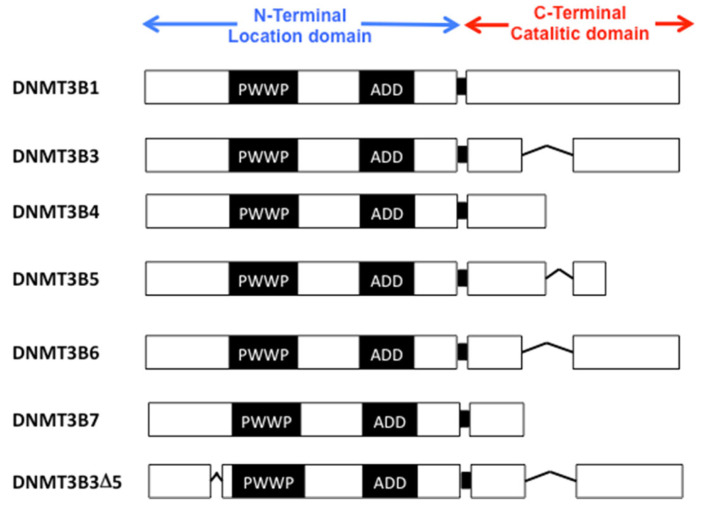
Structure of Human DNMT3B isoforms. Splice variants isoforms of DNMT3B have different characters. DNMT3B isoforms variates mammalians. Amino and carboxyl-terminal domains variates. DNMT3B isoforms’ nuclear location variates during embryonic development and in tissue specificity. Moreover, it has been found that DNMT3B isoform variates in cancer with their own origin tissue. Suggesting that DNMT3B isoforms have an important role in cancer development. Abbreviatures: motif of interaction with pro-trp-trp-pro (PWWP), and ATRX, DNMT3, DNMT3L domain (ADD).

DNMT3B isoforms could be classified into three families. The first includes the isoforms DNMT3B1-7, which are the better studied. DNMT3B1 and DNMT3B2 are the catalytically active isoforms of DNMT3B; although DNMT3B2 lacks exon 10 [100] (Figure 2), it is known that it is a catalytically active isoform [18]; however, DNMT3B3-7 are the catalytically inactive isoforms of DNMT3B. Differential expression of DNMT3B isoforms is necessary to regulate gene expression through cellular differentiation. Next to fertilization, catalytically active DNMT3B1 is downregulated, and catalytically inactive DNMT3B3 is overexpressed, suggesting a dynamic role in DNMTs isoforms [101]. In the next section, we will review the knowledge of each DNMT3B isoforms, its associated function, and studies that prove the importance of isoforms expression, mainly by pluripotent stem cells and cancer cells [102,103]. 

DNMT3B3 is a DNMT3B isoform that lacks exons 10, 21, and 22. But, it still possesses the methyltransferase motifs I, IV, VI, IX, and X, the target recognition domain, and 9 amino acids of motif XI in the carboxylic domain (Figure 2). DNMT3B3 does not methylate in vitro substrates but regulates DNMT3B1 to diminish cell DNA methylation. DNMT3B4 does not have the motifs IX and X that are important in catalytic activity (Figure 2). DNMT3B4 is the main isoform that functions as a negative DNA methylation regulator [13,104]. DNMT3B4 induces cellular arrest [105] and hypomethylation of the pericentromeric region as a cellular mechanism to ensure correct chromosome segregation [104,106]. DNMT3B5 and DNMT3B6 have not been studied in cells; however, it is known that they have a role in inhibiting DNMT3B. DNMT3B6 lack exon 10 and gain 12 amino acids in the amino domain compared with DNMT3B1 (Figure 2). DNMT3B6 is poorly associated with the global frequency of DNA methylation [107]. DNMT3B7 decreases cellular proliferation and increases p21 protein levels [105,108]. It is overexpressed in breast cancer [109] and has a role in inducing migration and differentiation [108,110].

ΔDNMT3B is the second family of DNMT3B isoforms. This family has seven members reported. They do not have the first exons before the PWWP domain (Figure 2). For example, ΔDNMT3B4 is involved in aberrant DNA methylation patterns in cancer, and it is overexpressed in epithelial hyperplasia from lung cancer, suggesting that ΔDNMT3B overexpression predisposes to cancer [111]. On the other hand, The lack of PWWP domain and 21 and 22 exons characterize the third family of DNMT3B isoforms (Figure 2). This family of DNMTs is overexpressed in several cancer cell lines and pluripotent cells and has been associated with cellular proliferation by colony assays [112].

The alternative splicing process regulates DNMT3B to produce several isoform products. These products have a role in mediating DNMT methylation patterns. Each DNMT3B isoform has a cell-specific role in maintaining the optimal function of DNMTs in the tissue, and transcriptional deregulation could be a part of cancer development. Overexpression of each DNMT3B isoform has been associated with any cancer initiation, development, progression, or metastasis. Probably, Isoforms are defined by tissue origin, embryonic origin, and external factors by which cells are exposed (Table 3). Therefore, DNMT3B isoform regulation could be used as biomarkers in diagnostic and prognostic treatment even combined with other DNMT regulatory mechanisms. For example, DNMT3B3 is ubiquitously expressed in the body tissues [18,106], and its overexpression has been associated with liver cancer, cirrhosis, and chronic hepatitis [104]. DNMT3B4 is also overexpressed in the presence of *Helicobacter pylori* infection. DNMT3B4 is also overexpressed in hepatitis, cirrhosis, and liver cancer cells [104]. On the other hand, DNMT3B7 is overexpressed in breast cancer [109], and overexpression of the ΔDNMT3B family has been reported in nonsmall lung cancer cells [113,114].

### 2.4. DNMTs in Embryonic Development

The variation of DNA methylation is essential in embryonic development. In fact, mutation of the catalytic domain of DNMT3B produces the syndrome of immunodeficiency facial instability 1 (ICF1) [118]. The expression of DNMTs is involved in embryonic development [99]. And the variation of the expression of DNMTs in the taxa is essential for embryonic development [119]. Variation of DNA methylation in different taxons, in different stages of development, and in the various tissues of individuals is an important question in the biology of DNMTs [120]. 

Importantly, it has been proved that germline mutation in the catalytic domain of DNMT3B has, as a consequence, the ICF1 syndrome in humans [118,121]. Then, the Knockout of DNMT3A/B in the murine model has lethal consequences [122]. In addition, in other invertebrate models (in arthropods, fishes, and mammals), it has been proved that the variation of DNA methylation, and the expression of DNMTs in animals, are both involved in embryonic development. DNA methylation varies depending on the development stage and in the taxonomic group. However, this affirmation is not valid for all animals with no DNA methylation detected (Table 2). Recent studies in DNA methylation, and embryonic development, have shown some examples of how DNA methylation depends on the stage of development of the specific taxon. For example, Medaka fish (*Oryzias latipes*) have shown that DNA demethylation in sperm cells is during maturation. However, DNA methylation of the cells shows a gradual de novo methylation, after fertilization, until the finish of the gastrula stage. In the same work, DNMT3B was measured, and they found that the decrease in DNMT1 and increase in DNMT3BB.1 are associated with DNA methylation patterns. In Zebrafish, the authors show that the phenomena are similar but have dramatic de novo methylation from late morula to gastrula. In mammalians, at this point, there are variations too. In a mouse model (*Mus musculus*), during maturation, sperms gain DNA methylation. After fertilization, the demethylation process starts until the blastocyst stage, and it occurs dramatically de novo methylation, compared with humans (homo sapiens) [120,123]. 

In contrast, human sperm DNA is demethylated during maturation and fertilization; the zygote demethylates until blastocyst and then starts the de novo methylation process [123]. These works, in development, show that DNA methylation and DNMTs variate during the different stages of life in the diversity of taxa. DNMTs quantity marks change in cells during development stages, tissues, and taxa. 

### 2.5. DNA Methyltransferases Are Regulated by Chemical Compounds and ncRNAs

**Figure 3:**
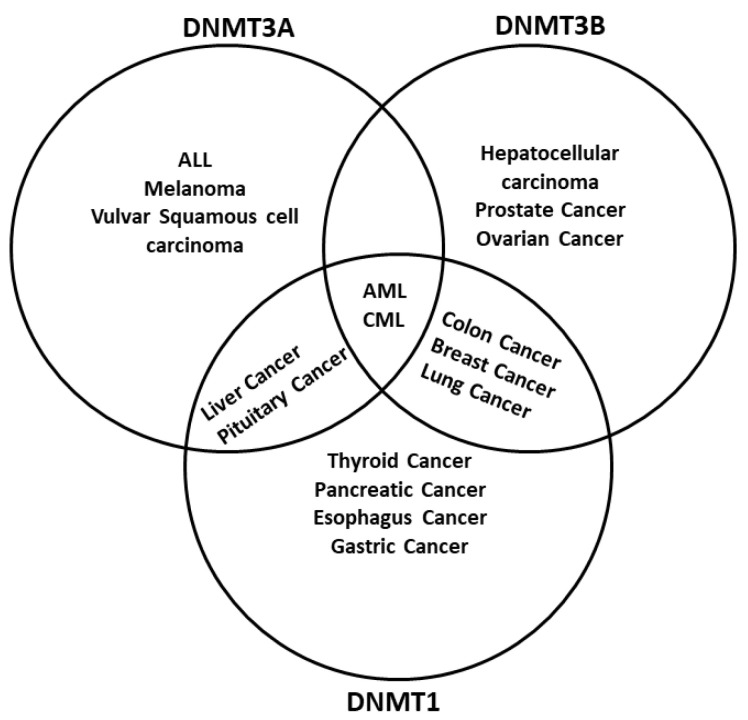
DNA methyltransferases are altered in cancer. DNMTs have a role in genomic regulation. In cancer, DNMTs are affected in expression level. In acute myeloid leukemia (AML) and chronic myeloid leukemia (CML), The three DNMTs are overexpressed. DNMT1 and DNMT3A have been described as affected in liver cancer and pituitary cancer, while DNMT1 and DNMT3B are overexpressed in breast cancer, colon cancer, and lung cancer; DNMT3B is deregulated in colon cancer and prostate cancer, and DNMT1 is deregulated in the pancreas cancer and esophagus cancer. In other cases, only one DNMT is overexpressed; however, only one DNMT could be enough to result in cancer development, progression, and metastasis. Abbreviatures: Myelodysplastic syndrome (MDS), chronic myelomonocytic leukemia (CMML), acute lymphoblastic leukemia (ALL), melanoma.

The dysregulation of DNA methylation has a role in the development of cancer cells and other diseases [7,124]. As DNA methylation is a critical factor in global epigenetic regulation [125], it is not surprising to find DNMTs dysregulated in cancer. Actually, one of the main epigenetic characteristics in cancer is the global demethylation and local hypermethylation of the DNA [126]. There are multiple examples of how different tumors have either a misregulation of one or more DNMTs or even mutations. For example, hematological diseases like acute myeloid leukemia (AML) have mutations in the *DNMT3A* gene [127], whereas inactivating mutations in *DNMT1* are related to genome-wide alterations of DNA methylation in colon cancer [128] (Figure 3). Different regulators of DNMTs have been researched, such as non-coding RNAs (ncRNA) and artificial compounds tested to influence DNA methylation. Targeting DNMTs is a promising tool to use alone or in combination to treat cancer. However, further research needs to be done in this field.

There are still only a few epigenetic drugs approved to treat cancer. One of the biggest challenges nowadays for clinical chemical treatments is to have greater efficiency, stability, and minimal adverse effects; the development of oligonucleotides and the discovery and synthesis of new drugs capable of inhibiting the expression of the DNMTs are becoming more used, and useful tool. The FDA has approved only two nucleoside analogs: 5-Azacytidine (Aza) and 5-aza-2′-deoxycytidine (DAC or decitabine). These compounds are used for treating myeloid dysplastic syndrome (MDS) and acute myeloid leukemia (AML) [129,130,131]. However, other molecules are currently being tested as possible therapy drugs in pre-clinical studies, like zebularine, a nucleoside analog more stable than Aza and DAC [132]. More recently, the second generation of decitabine and deoxyguanosine has been launched: Guadecitabine or SGI-110, which has proved to have a longer half-time thanks to being less prompt to deamination [9,133]. The DNMT inhibitor 5-fluoro-2′-deoxycytidine (FdCyd) has shown promising results in clinical trials. [129,130]. 

More recently, a series of new quinoline-based inhibitors have been discovered. These belong to the non-nucleoside inhibitors, the first of them is SGI-1027 [134], from which two other analogs have been described: MC3343 [134] and MC3353 [135]. For a more detailed revision of other molecules being studied as inhibitors of DNMTs, revise Hu et al., 2021 [136]. Until now, novel therapies do not only include DNMTs inhibitors but are also looking forward to including immunotherapy and HDACs inhibitors [137].

On the other hand, it has been reported that ncRNAs, which comprise small interfering RNAs (siRNAs), microRNAs (miRNAs), and long non-coding RNAs (lncRNAs), are capable of inhibiting DNMTs (Table 4). Firstly, siRNAs and miRNAs are small ncRNAs (siRNAs are 21–23 nucleotides, and miRNAs are 19–25 nucleotides) that silence gene expression at the post-transcriptional level by targeting the messenger RNA (mRNA). Secondly, lncRNAs have a size of more than 200 nucleotides, and they have a role in regulating gene expression at transcriptional and post-transcriptional levels [138]. Several examples of these molecules have been reported, such as MG98, a siRNA inhibitor of DNMT1. This has been employed in phases one and two of clinical trials with mixed results [139,140,141], suggesting that more research is needed to find the proper working doses, or even adequate combinations, to ensure better performance.

On the same line, miR29b is a miRNA that targets DNMT3A, and DNMT3B is downregulated in cancer of lymphoma and cancer of the pancreas [142,143]. In pancreatic cancer, it was shown that the expression of mir29b was downregulated; meanwhile, DNMT3b was enhanced. miR-29b overexpression caused a decrease in cell viability and promoted apoptosis by targeting DNMT3b. Meanwhile, in acute myeloid leukemia, the ectopic expression of miR-29b caused a reduced expression of DNMT3A and DNMT3B at both RNA and protein levels. This was because these DNMTs are direct targets of miR-29b [143]. In addition, miR-145 targets DNMT3A in ovarian carcinoma. The knockdown of DNMT3A decreases DNA methylation in the promoter of miR-145. This increased expression of miR-145 further downregulates DNMT3A, which is a double-negative feedback loop [144]. In bladder cancer, the lncRNA DBCCR1-003 directly interacted with DNMT1, preventing it from methylating the promoter region of the tumor suppressor gene DBCCR1 [145]. In AML, it has been reported that when *CCDC26* (a lncRNA that interacts with DNMT1) is lost, DNMT1 is mislocalized to the cytoplasm, turn causes the hypomethylation of the DNA [146]. Other lncRNAs that interact with DNMT1 and affect methylation are *DACOR1* in colon cancer [147] and *HOXD-AS1* in lung adenocarcinoma [148]. linc-POU3F3 also downregulates the expression of DNMTs in esophageal squamous carcinoma [149]. Although all of these molecules are potential tools in cancer treatments, only MG98 and miR-29b have been tested as therapy inhibitors of the DNMTs and further research needs to be done. However, it is useful to know that DNMTs also have ncRNAs to regulate them more specifically.

Cancer cell development has been associated with epigenetic alterations, and DNMTs are one of the main actors in transcription regulatory processes. DNMTs are regulated by several mechanisms as ncRNAs and deregulation of DNMTs have been found in cancer. These deregulations have been reported in several types of cancer in a combinatorial manner. That is why the regulatory mechanisms of DNMTs are important in the cancer study. However, studying not only the mechanism of action of the DNMTs but also their broad diversity in organisms and how they have evolved in time allows us to understand better how they are regulated and predict the consequences of mutations and deregulations. Furthermore, manipulating DNMTs expression could be of great importance to knowing the drugs and the different ncRNAs that can help regulate them in cancer cells.

## 3. Conclusions

Our understanding of the family function of DNMTs has increased considerably as their discovery about thirty years ago. It is now clear that these proteins play critical roles in setting DNA methylation patterns genome-wide at specific developmental time points, particularly during early development and in specific tissues undergoing dynamic methylation in living beings. Most recent studies indicate that DNA methylation plays a role in variation during evolutionary context and adaptation to the ambient conditions. Other studies have shown their importance in clinical and industry research. Future biochemical and structural studies should focus on DNMT and DNMT isoform-specific and their relation with ambient variables to assess phenotypic association and elucidate functions in different types of cells, parasites, fungi, plants, and animal models. More studies are also necessary to understand the role of DNMTs isoforms in cancer and its interrelation between tumors and the origin tissues.

## Figures and Tables

**Table 1:** DNA methyltransferases (DNMTs) in the five kingdoms.

Orthologs	DNA Methyltransferases Know in Each Taxonomic Group	Paralogs of DNA-Methyltransferases	Function Associated with Each Taxonomic Group	References
Monera	*Alphaproteobacteria*	5mC-MTase.	Restriction modification (RM), bacteriophage’s sequence silence	[36]
Protists	*Diatomeas* and *Choanoflagellates.*	MET1, CMT3, and DRM2.	Not experimentally probed	[37]
Algae	*Chlorella*, spp., *Clamydomonas*; *Volvox carteri.*	MET1, CMT1 and 3, and DMR.	Genomic imprinting and development	[38,39]
Fungi	*Humicola grisea*; budding yeast; *Neurospora* sp.; *Aspergillus clavatus.*	DIM-2, Masc1 and 2, and RID.	Genomic repression.	[40,41,42]
Plantae	*Arabidopsis thaliana*	MET1, CMT, DMR DNMT1, 2, and 3.	Genomic imprinting, reproduction, and development.	[25,43]
Animals invertebrates	*Nematoda*, *Equinoderma*, *Paracentrotus lividus*, *Solea seleganensis*, *Gadus morhua*, *Arthropoda*	DNMT1 and 3	Repetitive sequence repression, Development, Gene regulation, and epigenetic memory.	[44,45]
Animals vertebrates	Fishes and mammals	DNMT1, 2 and 3	Repetitive sequence repression, Development, Gene regulation, epigenetic memory, and Cancer progression.	[3,46,47]

**Table 2:** Species without DNA methylation detected.

Orthologs within DNA Methylation Detected	DNMT Reported In Specie	References
*Saccharomyces cerevisiae,*	-	[34]
*Caenorhabditis elegans*	-	[34,35,45]
*Drosophila melanogaster*	DNMT2	[31,32,33]

**Table 3:** Messenger RNA of DNMTs biomarkers.

DNMT3B Isoform	Type of Biomarker	Type of Pathology	References
DNMT3B3	Diagnostic	Cirrhosis Gastric cancer Hepatitis Liver cancer Ovarian carcinoma	[104,107,115]
DNMT3B4	Diagnostic	*Helicobacter pylori infection* Renal cancer	[13,104,105,116]
DNMT3B7	Diagnostic	Breast cancer	[105,108,109,110,117]
ΔDNMT3B4	Diagnostic	Hyperplasia Lung cancer	[111]

**Table 4:** Studies of DNA methyltransferases (DNMTs) and non-coding RNAs in cancer.

ncRNAs	DNMT Deregulated in Cancer	Type of Cancer	Type of Deregulation	Reference
DBCCR1-003	DNMT1	Bladder	Down	[145]
linc-POU3F3	DNMT1, 3A, and 3B	ESCC	Up	[149]
miR-148a	DNMT1	Gastric	Down	[150]
miR-29a	DNMT1	Liver	Down	[151]
miR-152	DNMT1	Glioma	Down	[152]
miR-185	DNMT1	Glioma	Down	[153]
miR-145	DNMT3A	Ovarian	Down	[144]
miR-101	DNMT3A	Glioma	Down	[154]
miR-29	DNMT3B	Burkitt	Down	[142]
miR-29b	DNMT3B	Lymphoma, pancreatic, head and neck cell line cancer	Down	[143,155]

## Data Availability

Not applicable.
